# Thyroid Screening in Early Pregnancy: Pros and Cons

**DOI:** 10.3389/fendo.2018.00626

**Published:** 2018-10-25

**Authors:** Peter N. Taylor, Stamatios Zouras, Thinzar Min, Kalyani Nagarahaj, John H. Lazarus, Onyebuchi Okosieme

**Affiliations:** ^1^Thyroid Research Group, Systems Immunity Research Institute, Cardiff University School of Medicine, Cardiff, United Kingdom; ^2^Endocrinology and Diabetes Department, Prince Charles Hospital, Cwm Taf University Health Board, Merthyr Tydfil, United Kingdom; ^3^Endocrinology and Diabetes Department, University Hospital of Wales, Cardiff, United Kingdom

**Keywords:** screening, pregnancy, hypothyroidism, hyperthyroidism, thyroid, obstetric, development

## Abstract

Universal thyroid screening in pregnancy is a key debate in thyroidology and obstetrics. It is well-established that thyroid hormones are essential for maintaining pregnancy and optimal fetal development. Thyroid dysfunction is common in women of child-bearing age and also results in substantial adverse obstetric and child neurodevelopmental outcomes. Furthermore, thyroid dysfunction is readily diagnosed with reliable blood tests and easily corrected with inexpensive and available treatments. Screening only high-risk patients appears to miss the majority of cases and economic models show that compared to high-risk screening, universal screening is cost effective even if only overt hypothyroidism was assumed to have adverse obstetric effects. As a result, several countries now implement universal screening. Opponents of universal thyroid screening argue that asymptomatic borderline thyroid abnormalities such as subclinical hypothyroidism and isolated hypothyroxinemia form the bulk of cases of thyroid dysfunction seen in pregnancy and that there is a lack of high quality evidence to support their screening and correction. This review critically appraises the literature, examines the pros and cons of universal thyroid screening using criteria laid down by Wilson and Jungner. It also highlights the growing evidence for universal thyroid screening and indicates the key challenges and practicalities of implementation.

## Introduction

Thyroid hormones are essential for maintaining pregnancy and optimal fetal development (1, 2). It is well-established that overt thyroid disease is associated with adverse obstetric and offspring neuro-developmental outcomes (1, 3). More recently there has been growing concern that more marginal degrees of thyroid dysfunction particularly subclinical hypothyroidism (elevated TSH and normal FT4 concentration) and isolated hypothyroxinemia (normal TSH and low FT4) are also associated with fetal loss, prematurity and impaired offspring cognitive function (4–6). In some studies, maternal thyroid autoimmunity has also been identified as a potential risk for fetal loss (1).

Because thyroid disorders are particularly common in women of reproductive age, thyroid dysfunction is frequently encountered during pregnancy, sometimes as a new diagnosis (7, 8). The prevalence of hypothyroidism is about 2% in iodine sufficient areas while overt and subclinical thyrotoxicosis occur in ~0.2 and 2.5% of pregnancies, respectively (9). Such thyroid disorders are frequently asymptomatic or difficult to distinguish from the features of normal pregnancy on clinical grounds alone. Thus, it would seem logical to systematically screen pregnant woman for thyroid disorders. However, such a screening strategy is likely to predominantly identify women with subclinical thyroid disease for whom the benefits of systematic screening and correction remain controversial (10, 11).

These competing considerations have fuelled continued debate on the merits of gestational thyroid screening (12). While universal thyroid screening is recommended in countries such as Spain (13), China (14), and Poland (15), other countries including the United Kingdom and the United States adopt a case-finding approach targeted at women at high-risk of thyroid dysfunction (16). In 1968, James Wilson and Gunner Jungner published their classic report on the principles and practice of screening in which they set down a number of pertinent criteria that should be considered before a screening programme is adopted (Box 1) (17). These criteria have since been widely applied to screening decisions and have stood the test of time as a gold standard tool for screening policies. In this review, we appraise the potential benefits and drawbacks of universal thyroid screening in early pregnancy using the Wilson and Jungner criteria (17) (Box 1).

Box 1 **Screening criteria.**

1. Is the condition an important health problem?
2. Is there an accepted treatment?
3. Are facilities for diagnosis and treatment readily available?
4. Is there a recognizable latent or early symptomatic stage?
5. Is there a suitable test or examination?
6. Is the test acceptable to the population?
7. Is the natural history of the condition, adequately understood?
8. Is there an agreed policy on whom to treat?
9. Is the cost of case-finding economically viable?
10. Case-finding should be a continuing process and not a “once and for all” project.

Adapted from Wilson and Jungner (17).

## Criteria 1: is the condition an important health problem?

### Pros

It is well-established that overt thyroid dysfunction is an important health condition in pregnancy. Overt hypothyroidism occurs in ~0.2–0.6% of pregnant women (2, 18) while overt hyperthyroidism, usually due to Graves' disease, occurs with a frequency of about 0.2% (3). More modest abnormalities of thyroid function are more prevalent. Subclinical hypothyroidism occurs in 2–3% of pregnancies (1, 16) and the prevalence of isolated hypothyroxinemia, defined as a normal TSH with FT4 below the 2.5 percentiles occurs in around 2% of pregnancies (19). In early gestation, fetal reliance on maternal thyroxine delivery coincides with a period of critical developmental landmarks such as neuronal proliferation, migration, and neural tube formation (20). Thus, maternal thyroid dysfunction in early pregnancy may have permanent repercussions on child neurodevelopment as exemplified in the devastating neurological sequelae of uncorrected congenital hypothyroidism or severe iodine deficiency (21).

In addition, observational studies show that offspring of women with hypothyroidism (22) or isolated hypothyroxinaemia (23), suffer with ~2–7-point deficits in IQ compared to children of euthyroid mothers (24). Two RCTs, the Controlled Antenatal Thyroid Screening (CATS) study (10), and a United States National Institutes of Health study by Casey et al. (11), have investigated the impact of systematic screening and correction of maternal subclinical thyroid dysfunction on child intellectual function. These studies showed no benefits of maternal levothyroxine on child IQ when evaluated at age 3 years (10) and 9 years (25) in the CATS study, and at 3–5 years in the study by Casey et al. (11). In both studies however, levothyroxine was initiated at a median gestational age of 13–18 weeks which would have been after the critical neurodevelopmental period and therefore possibly too late to have had an impact on fetal brain development.

Women with thyroid dysfunction also suffer an increased risk of adverse pregnancy outcomes an association which is well-established for overt thyroid dysfunction but is also seen with subclinical hypothyroidism (26). One meta-analysis showed significant risks of miscarriages (odds ratio 1.93, 95% confidence interval 1.4, 2.64) and pre-term loss (odds ratio 1.3, 95% confidence interval 1.05, 6.0) in women with subclinical hypothyroidism (6). Two small fertility clinic trials in women undergoing assisted reproduction techniques also reported beneficial effects of levothyroxine in increasing clinical pregnancy and live birth rates in women with subclinical hypothyroidism (27, 28). In a prospective controlled trial, systematic screening and correction of gestational subclinical hypothyroidism significantly reduced adverse pregnancy events in the sub-group of women classified as low risk for thyroid disease (29).

### Cons

The health impact of overt thyroid dysfunction is not in doubt but the bulk of cases of thyroid dysfunction that will be detected by systematic screening in pregnancy will be asymptomatic subclinical or borderline biochemical abnormalities. For example, subclinical hyperthyroidism, in which TSH is low in the presence of normal FT4, is usually due to the transient thyroid stimulatory effects of human chorionic gonadotrophin (hCG) and does not require treatment (30). Also, the significance of subclinical hypothyroidism or isolated hypothyroxinaemia and the benefits of correcting these biochemical abnormalities has not been proven in RCTs (10, 11). While intervention in controlled trials were initiated relatively late it would be challenging to achieve earlier treatment in routine clinical practice.

## Criteria 2: is there an accepted treatment?

### Pros

Levothyroxine is a safe and well-tolerated treatment for hypothyroidism (7). There is extensive data on its pharmacokinetics and over 50 years of experience with the synthetic form of the drug (31). Other preparations such as liothyronine and desiccated thyroid extract are not recommended in pregnancy due to safety concerns (16). In symptomatic patients with overt hypothyroidism Levothyroxine leads to rapid improvement in well-being and has been shown to improve pregnancy outcomes (26). Inadequate levothyroxine correction may also lead to pregnancy loss even in women established on treatment (32). The thionamide compounds, Methimazole, its pro-drug derivative, Carbimazole, and Propylthiouracil are the mainstay of treatment for hyperthyroidism in pregnancy. While these compounds are associated with a number of rare side effects, including embryopathy and maternal liver failure (33), they are generally safe and well-tolerated, with benefits that outweighs the risk of fetal harm from drug side effects.

### Cons

Although Levothyroxine is the accepted treatment for overt hypothyroidism its benefits in gestational subclinical thyroid dysfunction remains uncertain. More so, the use of Levothyroxine is not without the risk of over-treatment especially in women with borderline abnormalities. Studies have shown a high proportion of over-treatment in levothyroxine users ranging from 20 to 40% in the general population (31), and 10% during pregnancy in the CATS study (10). Korevaar et al. have shown an inverted U-shaped association between maternal T4 and child IQ with IQ deficits of 1.4–3.8 points at the extremes of T4 (34). Thus, inappropriate use of Levothyroxine could potentially do more harm than good.

## Criteria 3, 5, 6: are facilities for diagnosis and treatment readily available, is there a suitable test or examination and is the test acceptable to the population?

### Pros

The diagnosis of thyroid dysfunction is established by measurement of the thyroid hormones FT4, FT3, and TSH (16). In addition, thyroid peroxidase antibodies (TPOAb) and TSH receptor antibodies (TRAb) may be tested to confirm autoimmunity which is the commonest cause of thyroid dysfunction in iodine-replete countries. These simple non-invasive tests are relatively inexpensive and available in modern laboratories. Pharmacological treatments for thyroid dysfunction are easily accessible and guidance for their use and monitoring is well-established. In pregnancy, it is recommended that each laboratory derive its own trimester specific reference range using a local population of thyroid disease-free pregnant women (16, 35). In the absence of local data, reference ranges may be extrapolated from a population with similar ethnicity, iodine nutrition, and assay methods (1, 16) or as suggested by the current guidelines of the American Thyroid Association (ATA), the TSH reference range in pregnancy may be set at 0.5 and 0.4 mU/L below the upper and lower non-pregnant limits, respectively (16).

### Cons

The assessment of thyroid function in pregnancy is by no means straightforward and is subject to misinterpretation due to the range of physiological adaptations that maintain fetal thyroid hormone delivery in the face of increased thyroid hormone requirements (36). These changes, including increased thyroxine binding globulin production, stimulation of the TSH receptor by hCG, and increased peripheral thyroid hormone metabolism, all have implications for the evaluation of thyroid function tests in pregnancy (37). Although gestation specific reference ranges are recommended, many centers use the general population reference ranges or continue to use the 2.5/3.0 mU/L threshold which would lead to over-diagnosis. Furthermore, the pregnancy-specific reference ranges in use are typically derived from cross-sectional samples that do not account for differences in longitudinal trajectories between healthy and autoimmune thyroid disease states as demonstrated in recent studies (38, 39).

## Criteria 4, 7: is there a recognizable latent or early symptomatic stage and is the natural history of the condition understood?

### Pros

Subclinical hypothyroidism and thyroid autoimmunity may represent early-disease states in the evolution of hypothyroidism. Subclinical hypothyroidism is present in 4–10% of the general population (40) and progresses to overt hypothyroidism at a rate of about 2–5% per annum or higher in the presence of antibodies (41). In pregnancy, about 25% of women with subclinical hypothyroidism will have persistent TSH elevation following delivery (42). Positive thyroid antibodies in euthyroid women, i.e., euthyroid autoimmunity, is seen in about 10% of pregnant women (43), a fifth of whom will develop hypothyroidism in the course of pregnancy (44). The obstetric consequences of euthyroid autoimmunity remain controversial but an increased risk of pregnancy loss has been observed in meta-analyses of spontaneous and assisted pregnancies (45, 46). Possible mechanisms for such pregnancy loss include intrinsic thyroid hormone deficiency, diffuse autoimmunity, and impaired thyroidal response to hCG (3, 39, 47).

### Cons

It can be difficult, without the appropriate expertise, to reliably distinguish pathological thyroid disease from non-significant fluctuations in thyroid hormones. Although subclinical disease may precede overt disease, not all patients with subclinical disease develop overt disease. More importantly, the wealth of observational data showing an increased risk of adverse pregnancy outcomes in women with subclinical thyroid dysfunction are not reconciled with data from randomized controlled trials. Thus, targeting subclinical thyroid disease in pregnancy will inadvertently promote overdiagnosis and fuel unwarranted clinician and patient anxiety.

## Criteria 8. is there an agreed policy on whom to treat?

### Pros

All society guidelines endorse the treatment of overt thyroid disease and in addition, most recommend treating antibody-positive women with subclinical hypothyroidism (16, 48, 49). The approach to euthyroid autoimmunity or women with subclinical hypothyroidism and negative antibodies is less consistent (16, 50). As with other screening programmes shared treatment decisions can be reached with patients following a fully informed discussion of potential risks and benefits.

### Cons

Navigating the various diagnostic gray areas could be challenging for general clinicians and patients alike. An individualized approach is reasonable but will be difficult to administer outside of special-interest units. Ultimately, frontline clinicians may be overwhelmed by demands for treatment from an increasingly health-seeking population.

## Criteria 9 and 10. is the cost of case-finding (including diagnosis and treatment of patients diagnosed) economically viable? case-finding should be a continuing process and not a “once and for all” project

### Pros

Current international guidelines recommend screening for thyroid dysfunction in women at high risk of thyroid dysfunction, i.e., case-finding. However, various antenatal clinic studies have consistently shown that case-finding fails to diagnose 30–80% of women with hypothyroidism (51–54). An economic analysis using a state transition Markov model compared the cost-effectiveness of universal screening, comprising first trimester TSH and TPOAb measurements, case-finding, and no-screening (55). Of the three strategies, universal screening was the most cost-effective with an incremental cost-effectiveness ratio of 7258 USD per quality-adjusted life year (QALY) over risk-based screening. Importantly, universal screening remained cost–effective if the model assumed that only overt hypothyroidism was associated with adverse obstetric outcomes with a cost-effectiveness ratio of $7335/QALY over risk-based screening (55). In another study from Spain the authors estimated that use of universal screening over high-risk screening would yield annual savings of €2,653,854 for the Spanish National Health System with an incremental cost-effectiveness ratio of €374.28/QALY over no screening (56). These figures compare favorably to the cost-effectiveness of gestational diabetes mellitus screening ($12,078/QALY) in the United States for example (57) and is well below the $50,000/QALY threshold for which a screening intervention is considered value for money.

### Cons

The analysis by Dosiou et al. was based on two RCTs conducted in Southern Italy, an area of mild iodine deficiency. There is no guarantee that the benefit reported in these studies will be applicable to countries with different iodine nutrition or resource settings. Furthermore, the overall costs of establishing a universal screening programme will need to be carefully considered and will need to incorporate the costs of personnel training, public education, establishment of healthcare infrastructure, and the burden of over-diagnosis and over-treatment.

## Discussion

In this appraisal, we have examined the pros and cons of universal thyroid screening in early pregnancy using the template set by Wilson and Jungner. Our analysis presents a number of compelling arguments in support of universal screening but also raises areas of uncertainties and practical caveats. Universal thyroid screening in pregnancy fulfills most of the criteria for screening (Table 1). Thyroid disease is an important health problem in pregnancy with acceptable and well-established treatments. Diagnostic facilities are available and relatively inexpensive and there is adequate knowledge on the natural history and impact of thyroid disorders on feto-maternal well-being. Screening all pregnant women with TSH and TPOAb in the first trimester of pregnancy is cost-effective compared to targeted or no screening at all and benefits are apparent even if only overt hypothyroidism was considered (55). Although the merits of treating maternal subclinical thyroid dysfunction have not been proven in RCTs it is probable that treatment in current RCTs was started too late in pregnancy to be effective (10, 11).

**Table 1:** Appraisal of universal thyroid screening in pregnancy based on the Wilson and Jungnen criteria.

**Criteria**		**Hypothyroidism**	**Hyperthyroidism**	**Comments**
1	Importance	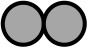	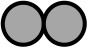	Common condition with established adverse feto-maternal effects
2	Accepted treatment	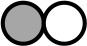	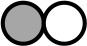	Accepted for overt disease but uncertain for subclinical thyroid dysfunction
3	Facilities available	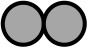	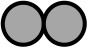	Widely available and relatively inexpensive
4	Latent period	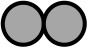	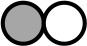	May be difficult to distinguish physiological from subclinical states
5	Suitable test	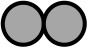	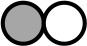	Need for local gestational age-specific reference ranges
6	Acceptable test	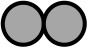	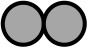	Acceptable and established
7	Natural history	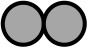	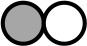	May be difficult to distinguish physiological adaptations from true pathology
8	Agreed policy	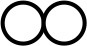	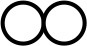	No consensus for subclinical thyroid dysfunction
9	Economically viable	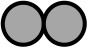	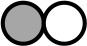	Cost-effective overall and for overt hypothyroidism only
10	Continuing Process	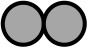	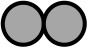	Screening is a continuing process

*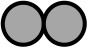, Fulfills criteria; 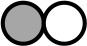, Partially fulfills criteria; 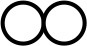, Does not fulfill criteria*.

Of all ten criteria, only criteria 8, “there should be an agreed policy on whom to treat as patients,” is not satisfied. This is understandable given that thyroid dysfunction, like some other conditions with screening programmes such as hypertension or dyslipidaemia, is a continuum in which the thresholds for intervention are uncertain. Whilst all societal guidelines endorse treatment of overt thyroid disease, the uncertainty lies in the management of subclinical hypothyroidism, isolated hypothyroxinemia, and euthyroid autoimmunity. Here, the current ATA guidance (16) to definitely treat women with TSH > 10 mU/L or antibody-positive women with TSH > 4.0 mU/L seems logical as such women almost certainly have intrinsic thyroid disease and will progress to thyroid failure. The less prescriptive advice in gray areas provides flexibility for shared clinician patient decisions in particular the fact that treatment can be considered in TPO antibody positive women with a TSH > 2.5 mU/l. More research is needed to understand the impact of euthyroid autoimmunity or isolated hypothyroxinaemia. Of interest will be the results of the TABLET trial, a large multicentre UK randomized controlled trial of Levothyroxine on pregnancy and neonatal outcomes in women with thyroid antibodies (58).

A number of practical considerations will need to be addressed before a universal thyroid screening can be safely implemented. First, the use of pregnancy specific reference ranges derived from a local population of thyroid disease-free pregnant women should be considered a priority, even a prerequisite for thyroid disease management in pregnancy. This will go some way in distinguishing true pathology from pregnancy induced physiological adaptation and will help curtail the dilemma of over-diagnosis. Second is the need to establish consensus criteria and unified nomenclature for the diagnosis of thyroid conditions in pregnancy, using definitions that are adaptable across populations and laboratories. The acquisition of skills in interpreting abnormal thyroid function tests in pregnancy by endocrinologists and obstetricians and the availability of resource, referral pathways, and well-defined inter-disciplinary responsibilities is essential.

The ultimate aim of maternal thyroid disease screening in pregnancy is to optimize feto-maternal outcomes. Thus, therapeutic intervention when indicated should be implemented as early as possible in the course of fetal development. Accordingly, universal screening should ideally be performed once pregnancy is suspected or pre-conception if pregnancy is planned. A pragmatic algorithm is to measure TSH and then reflex FT4 and TPOAb if TSH is outside of the relevant reference range. This will be challenging to achieve but with thoughtful planning can be integrated into routine community health services. An example of good practice in this regard is China's national free preconception health examination project which offers access to preconception health examinations including thyroid hormone measurement in rural parts of China (14). A recent analysis of 184,611 pregnant participants in this programme showed an association between higher preconception TSH concentrations and risks of miscarriages and pre-term loss, highlighting opportunities for modifying pregnancy outcomes with preconception care (59).

Most national societies recommend targeted case-finding, although with an expanding definition of high-risk (16). In practice there is wide variation ranging from case-finding to a near-universal or universal approach. For example, 42% of European endocrinologists reported that they routinely screen all women in pregnancy while 43% performed targeted high-risk case finding (60). In contrast 85% of women in routine antenatal clinics in Boston underwent screening (53). Several national societies now recommend universal screening programmes. The Polish Society of Endocrinology recommend measuring TSH either preconception or at 4–8 weeks gestation in all pregnant women (15). Likewise, the Spanish Society of Gynecology and Obstetrics recommend universal screening with TSH levels in the first 10 weeks of gestation and then FT4 if TSH > 2.5 mU/L (13). Continuous audit, review and refinement of these programmes will be essential for understanding the impact of screening.

Ensuring preconception iodine sufficiency through targeted public health campaigns and sufficient prenatal iodine supplementation should be integrated with thyroid screening programmes. Iodine deficiency is common during pregnancy, particularly in Europe (61) and correction of mild-moderate iodine deficiency may have obstetric and offspring benefits (62) although a recent large trial of iodine supplementation in pregnancy in mild iodine deficiency failed to show benefits on offspring IQ (63). However, in countries like the United Kingdom which are iodine deficient in pregnancy with no routine iodine fortification programs, iodine supplementation during pregnancy and ideally 3 months prior to conception is desirable (64). The United Kingdom position is surprising given the action already taken by most other high, middle and low income countries to have iodine fortification if they are also iodine deficient in pregnancy Iodine supplementation may also be of benefit to some high risk groups for pregnancy. Certain population groups are at increased risk of being iodine deficient. In particular, obese women are more likely to be iodine deficient and have other micro-nutrient deficiencies in pregnancy (65).

## Conclusion

Universal thyroid screening in pregnancy fulfills most criteria for a beneficial and cost-effective screening programme and holds promise for improving fetal and maternal outcomes. However, areas of uncertainty remain especially with regards to the significance of borderline biochemical abnormalities and whether correction of such abnormalities can improve outcomes. A consensus is unlikely to be reached without further controlled trials and such trials should aspire to recruit women pre-conception or as early as possible in pregnancy. In the interim regular audit of existing screening programmes will be crucial in gaining insights into the practicalities of universal thyroid screening in pregnancy. For centers undertaking universal or high-risk screening integrating thyroid auto-immunity into decision making is essential.

## Author contributions

PT, SZ, TM, and KN drafted the manuscript with supervision and guidance from JL and OO. OO substantially edited the manuscript. All authors approved the final version.

### Conflict of interest statement

The authors declare that the research was conducted in the absence of any commercial or financial relationships that could be construed as a potential conflict of interest. The reviewer EP declared a past co-authorship with one of the authors JL to the handling editor.
